# LATE-LIFE TIME-RESTRICTED FEEDING AND EXERCISE DIFFERENTIALLY ALTER HEALTHSPAN IN OBESITY

**DOI:** 10.1093/geroni/igz038.386

**Published:** 2019-11-08

**Authors:** Marissa Schafer, Daniel Mazula, Thomas White, Vesselina Pearsall, Zaira Aversa, Jordan Miller, Nathan LeBrasseur

**Affiliations:** 1 Mayo Clinic, Rochester, Minnesota, United States; 2 Robert and Arlene Kogod Center on Aging, Mayo Clinic, Rochester, Minnesota, United States

## Abstract

Aging and obesity increase multimorbidity and disability risk, and determining interventions for reversing healthspan decline is a critical public health priority. Exercise and time restricted feeding (TRF) benefit multiple health parameters when initiated in early-life, but their efficacy and safety when initiated at older ages are uncertain. Here, we tested the effects of exercise versus TRF in diet-induced obese, aged mice from 20 to 24 months of age. We characterized healthspan across key domains: body composition, physical, metabolic, and cardiovascular function, activity of daily living (ADL) behavior, and pathology. We demonstrate that both exercise and TRF improved aspects of body composition. Exercise uniquely benefited physical function, and TRF uniquely benefited metabolism, ADL behavior, and circulating indicators of liver pathology. No adverse outcomes were observed in exercised mice, but in contrast, lean mass and cardiovascular maladaptations were observed following TRF. Through a composite index of benefits and risks, we conclude the net healthspan benefits afforded by exercise are more favorable than those of TRF. Extrapolating to obese older adults, exercise is a safe and effective option for healthspan improvement, but additional comprehensive studies are warranted before recommending TRF.

